# What can public health communicators learn from Reddit? A perspective for the next pandemic

**DOI:** 10.3389/fpubh.2024.1348095

**Published:** 2024-04-08

**Authors:** Irina Bergenfeld

**Affiliations:** Hubert Department of Global Health, Emory University, Atlanta, GA, United States

**Keywords:** COVID-19, vaccines, infodemic, social media, participatory

## Introduction

COVID-19 emerged in a world in which vaccine hesitancy was already endemic (1). According to the Alma Ata Declaration, providing “immunization against the major infectious diseases” is one of governments' basic responsibilities to their citizens (2), yet national governments have often struggled to find effective and ethically sound strategies to improve vaccine uptake among the hesitant and vaccine-averse. In 2019, the World Health Organization warned that vaccine misinformation had become a leading threat to global public health (3). The politicization of the COVID-19 pandemic and of efforts to control it accompanied a flood of mis- and disinformation, which governments and the private sector failed to effectively counter. As a result, even in countries with adequate vaccine supply and relatively few barriers to access, vaccination rates lagged well behind available doses, and excess vaccines expired unused. Local governments began offering cash incentives to motivate resistant individuals to get vaccinated, to little effect (4) and much ethical debate about coerciveness (5). In the US, official sources of health information, including the White House, Centers for Disease Control and Prevention, and local health departments offered confusing and sometimes conflicting information on COVID-19 and the vaccine. In Europe, “over-zealous” national governments discontinued the use of a specific vaccine against World Health Organization recommendations, undermining confidence in its safety (6). This lack of coordinated, effective messaging around COVID vaccination compounded an existing dearth of trust in public institutions and served to further hinder demand creation for the vaccine (6, 7).

The World Health Organization outlines four key activities for infodemic management: (1) listening to community concerns and questions; (2) promoting understanding of risk and health expert advice; (3) building resilience to misinformation; and (4) engaging and empowering communities to take positive action (8). The COVID-19 pandemic underscored that current and future efforts to address vaccine hesitancy will require interdisciplinary and multisectoral approaches involving the coordinated efforts of institutions, healthcare providers, behavioral scientists, and the media. Knowledge transfer from public health institutions to the public will not be sufficient to create demand for a product as contentious as a new vaccine (6). Researchers who study vaccine hesitancy and acceptance have found that emotions dominate health decision-making around vaccines (9–11) and that narratives and tropes are often more influential than facts and statistics (12, 13). Loss-framed messaging emphasizing the potential risks of not getting vaccinated may be more impactful on behavioral intention than gain-framed messaging about vaccine benefits (14–16). Message framing may be especially crucial for newer vaccines, which are perceived as riskier than established ones (17). This is true across the world- in a large, qualitative study on vaccine demand creation among pregnant women in Kenya, several participants recalled a persuasive radio advertisement featuring a man paralyzed by polio who wished he had been vaccinated as a child (18). At the provider level, where directly addressing misinformation can increase hesitancy, active and empathetic listening is a key avenue for reaching vaccine hesitant individuals and their children (7, 19, 20). While social media companies have responded to misinformation by increasing fact-checking efforts and removing disinformation, these are often “too little, too late” (21, 22). As an alternative, multidisciplinary researchers have begun to explore the potential of “inoculating” social media users against misinformation by familiarizing them with the common tactics that shady actors use to promote false and misleading vaccine narratives (23). Still others have taken a bigger-picture view, emphasizing the importance of proactive efforts to improve vaccine literacy (24) and public trust in government (25) to lay a foundation for improving vaccine uptake.

## Participatory health communication to promote scientific literacy and combat misinformation: examples from the pandemic

Social media often served to amplify misinformation during the pandemic and to weaken confidence in vaccines (26), as anti-vax content was able to emerge and spread while official vaccine communication was still “getting its pants on,” so to speak (27). However, there were notable exceptions where private citizens leveraged social media to fill the public health communication gap. Unsurprisingly, jokes and memes have been found to be some of the most popular formats for both pro- and anti-vax messaging on social media (28). The viral success of Vick Krishna, whose humorous 2021 “Fork Hands” TikTok post explaining mRNA technology to lay audiences was viewed by millions, demonstrates the potential of social media approaches in promoting vaccine literacy (24) among the general population (https://www.npr.org/sections/goatsandsoda/2021/04/01/983397422/the-viral-tiktok-video-that-explains-vaccine-science-and-makes-you-laugh). Another example of the organic emergence of vaccine communication during the COVID-19 pandemic is the Reddit community, or *subreddit*, known as r/HermanCainAward, which reached over half a million members by the end of 2023. The subreddit features posts of a chronologically sequenced, anonymized screenshots of social media accounts that shared COVID or vaccine-related disinformation. Each post forms a narrative that follows the “nominee”'s social media posts from misinformation to hospitalization (and often death). It is possible to trace the ebb and flow of the pandemic itself in the r/HermanCainAward post history, with trends in traffic on the subreddit paralleling COVID surges. As the community's popularity grew, it sparked attention, criticism, and debate among traditional media outlets such as Fox News, VICE, and National Public Radio. A 2021 piece from *Slate* magazine presciently observes: “[The Herman Cain Award subreddit] is an anti-persuasive venue, a place that dispenses with rational appeals for people to behave better in favor of something much more primal and horrifying. And who knows? Maybe it's persuading people specifically because it's not trying to.” After the introduction of the vaccine, qualitative evidence of the subreddit's impact appeared in the rise of #IPA (Immunized to Protect against Award) posts, in which previously vaccine hesitant members photograph their vaccination cards as proof of their commitment to avoid becoming the next “nominee.”

Debate around the ethics of the Herman Cain Award, while rightly questioning the morality of public shaming, neglect the potential effectiveness of certain aspects of its approach. The online community adopted many of the recommended strategies for vaccine messaging, which official sources had largely neglected up to that point in the pandemic's trajectory. Firstly, Reddit's front page is visible to all users, regardless of their personal browsing history. This may be key to overcoming the siloing between anti-vax and pro-vax networks that occurs on other platforms, allowing messaging to reach a broad range of individuals across the spectrum of vaccine acceptance (29). Secondly, posts on r/HermanCainAward, taken straight from the source, are not burdened with the level of public distrust that increasingly plagues experts and public health institutions (30). The message content is produced by members of its target audience and curated by Reddit users to follow a simple narrative format, an example of the participatory approach to public health communication enabled by social media (31). Thirdly, messaging is strongly loss-framed, which may be necessary to overcome the increased risk aversion associated with decision-making around newer vaccines. Finally, r/HermanCainAward highlights the role that exposure to misinformation played in the deaths of awardees, and may therefore function similarly to inoculation strategies against false and misleading vaccine narratives (23).

## Discussion

This piece focuses on a single specific example of participatory vaccine communication on a single social media platform, limiting the conclusions that can be drawn. Reddit users are not necessarily representative of social media users generally, and content moderation works differently on Reddit than on many other sites. Moreover, defining roles and responsibilities of private social media companies in combating disinformation and promoting vaccination remains a contentious ethical issue (32, 33). Nevertheless, the Herman Cain Award presents an innovative approach to vaccine communication of which public health officials might consider adopting specific aspects: (1) narrative elements, (2) loss-framed messaging, (3) highlighting the dangers of disinformation, (4) knowledge co-creation, (5) and non-traditional partnerships and channels of dissemination. Indeed, 3 years into the pandemic, it seems that some institutions may be starting to test more effective strategies. For example, the White House has begun to recruit social media influencers to promote uptake for available COVID-19 vaccines, getting the message to those who might otherwise avoid pro-vaccine content (https://www.nytimes.com/2021/08/01/technology/vaccine-lies-influencer-army.html) (34). While such approaches do have their limits, leveraging personal social media accounts with broader audiences can be one of a range of strategies to reach individuals who might be less likely to trust more “official” sources of public health information. Where formal public health communicators still have a lot of work to do is the content and framing of their messaging. Large public health institutions still define their mission with regard to vaccine hesitancy as one of knowledge transfer to the public, assuming that simply conveying facts will be sufficient to change minds (and behaviors) when empirical evidence does not support this strategy (35). The marketing departments of pharmaceutical companies understand that their job is to create demand, and a compelling story is often a better strategy than an infographic. It is time for public health institutions to recognize that if they want to improve COVID-19 vaccination rates, they will need to provide more than facts and statistics and begin to leverage the tools of behavioral science, as advertisers do. Researchers have already found some success using strategies such as social listening (36) and knowledge co-creation (37) to combat vaccine misinformation. Producing coordinated, targeted, and narrative-based social marketing that makes the intended audience and their concerns feel heard will require public health institutions to form non-traditional partnerships and engage more reciprocally with the people they serve. Ultimately, if we are going to combat the flood of vaccine disinformation ahead of the next pandemic, we cannot be afraid to get our feet wet.

## Author contributions

IB: Writing – original draft, Writing – review & editing.
